# Examining Short Temporal Changes in Intertidal Macroalgal Microbiomes at 'Ewa Beach, O'Ahu, Hawai'i: Some Hosts Varied While Others Remained Stable

**DOI:** 10.1111/1758-2229.70333

**Published:** 2026-04-06

**Authors:** Evan S. Dunn, Heather L. Spalding, Kristina M. Hill‐Spanik, Heather Fullerton

**Affiliations:** ^1^ Department of Biology College of Charleston Charleston South Carolina USA; ^2^ Grice Marine Laboratory, College of Charleston Charleston South Carolina USA

**Keywords:** amplicon sequence variants (ASVs), community structure, macroalgae, similarity percentage analysis (SIMPER), tropical intertidal

## Abstract

Understanding the temporal variability of microbiomes is crucial for predicting dynamics within macroalgal communities under future climate change scenarios, rising temperatures, and increased marine heatwave events. Short‐term variation has been observed in human‐ and coral‐associated microbes, but these dynamics are less understood in macroalgae. Intertidal macroalgal communities are ideal systems for investigating microbiome temporal variation due to their exposure to daily fluctuations in abiotic conditions. We characterised and examined the variability in the microbiota of seven macroalgal species, with representatives from three different phyla, across five sequential low tides in May 2021 at a single intertidal bench at 'Ewa Beach, O'ahu, Hawai'i, USA. Bacterial community compositions found in two red algae, *Acanthophora spicifera* and *Laurencia dendroidea*, and one brown alga, *Dictyota sandvicensis*, had variable abundances of several amplicon sequence variants contributing to significant dissimilarity through time. Two green macroalgae ( *Avrainvillea lacerata* and *Halimeda discoidea* ) were stable over time. Temporal stability of the macroalgal microbiotas, therefore, was variable amongst macroalgal species, but may be dependent on its horizontal or vertical positioning within the intertidal zone, which can vary the level of environmental stress (e.g., temperature, light, desiccation). Additional work exploring the drivers of the temporal variability observed herein is needed.

## Graphical abstract

We examined microbiome variability in seven macroalgal species over 5 days at an intertidal bench in 'Ewa Beach, O'ahu, Hawai'i. Microbial communities of some red and brown algae fluctuated, whilst green algae remained stable. Temporal patterns varied by species and potentially by differences in intertidal positioning and exposure to environmental stress.

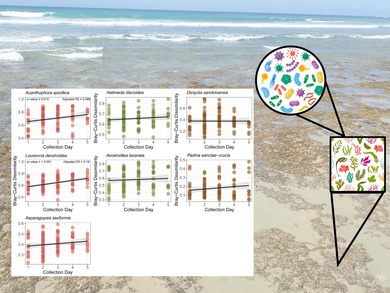

## 1 Introduction

Microorganisms colonise all surfaces and live both on and within organisms, thus forming their microbiome. The associated bacteria can be host‐specific and have symbiotic or pathogenic relationships (Case et al. 2011). Microbiomes associated with macroalgae have been shown to supply the host alga with CO_2_, vitamins like vitamin B_12_ (Grant et al. 2014; Bunbury et al. 2022), and morphologically important compounds (Matsuo et al. 2005; Ghaderiardakani et al. 2017, 2024). Microbiomes function as a defence for some macroalgal hosts via a high abundance of bacterial species with antimicrobial properties (Wiese et al. 2009), and may reduce bleaching disease and mitigate dysbiosis (Li et al. 2021, 2022). On the other hand, shifts in macroalgal microbiomes can cause diseases via invasion and infection of opportunistic bacteria (one or more) when the macroalgae are temperature‐stressed, such as with bleaching disease in *Delisea pulchra* (Campbell et al. 2011; Case et al. 2011). In turn, macroalgae provide microbes with sources of fixed carbon in the form of phylum‐specific cell wall polysaccharides, as well as a surface for settlement (Jung et al. 2013; Malik et al. 2019).

Being structurally complex, macroalgae can also influence microbial community composition through the production of secondary metabolites and oxidative bursts (Weinberger et al. 1999; Persson et al. 2011; Saha et al. 2011). Macroalgae are also phylogenetically diverse, classified into three phyla based on their characteristic pigments: Rhodophyta in class Florideophyceae, or red algae, contain chlorophyll *a* and phycobiliproteins (Mayanglambam 2015; Graham et al. 2022); Chlorophyta in class Ulvophyceae, or green algae, contain chlorophylls *a* and *b* as well as carotenoids (Lewis and McCourt 2004; Graham et al. 2022); and Heterokontophyta in class Phaeophyceae, or brown algae, contain chlorophylls *a* and *c* as well as fucoxanthin and several carotenoids (Terauchi et al. 2015; Graham et al. 2022). Cell wall composition varies amongst and within these phyla (Popper et al. 2011; Fuertes‐Rabanal et al. 2025) and thus may play a role in shaping their associated bacterial communities. Additionally, some macroalgae are calcified with CaCO_3_ on their surface (e.g., brown algae *Padina* Adanson), or within intercellular spaces as found in green algal genus *Halimeda* J.V. Lamouroux (Buapet and Sinutok 2021) and red algal genera *Corallina* Linnaeus and *Lithothamnion* Heydrich.

Short‐term dynamics in macroalgal microbiomes are important to understand because of the high diversity of macroalgae (16,506 described species (Guiry 2024) accessed on 08/10/2025) and their role as key ecosystem engineers (Jones et al. 1994). In the dynamic intertidal zone, macroalgae and their microbiota are impacted by both abiotic and biotic parameters. The macroalgal communities are shaped by fluctuations in abiotic stressors during tidal cycles (Doty 1946; Schonbeck and Norton 1979; Cox and Foster 2013; Cox et al. 2017; Piñeiro‐Corbeira et al. 2018; Ramos et al. 2020), as well as biotic factors like herbivory, competition, and microbial interactions. Herbivores may negatively impact algal distributions and abundances through grazing (Smith et al. 2002; Duran et al. 2018; Fowles et al. 2018), whilst epiphytic invertebrates can alter macroalgal microbiomes, leading to microbiota indicative of dysbiosis and pathogenesis (James et al. 2020). Competition, including interactions with invasive species (as defined by (Executive Order 13112—Invasive Species 1999) National Invasive Species Information Centre) of algae, also influences community structure and bacterial assemblages associated with macroalgae (Aires et al. 2013; Epstein et al. 2019; Silva et al. 2021). Bacterial communities can also change in structure over short periods (days), as seen with those associated with seagrasses (Wang et al. 2021) and corals (Ziegler et al. 2019; Caughman et al. 2021). Several experimental studies have shown that macroalgal associated microbial communities can change over the course of weeks or months following disturbances, such as transplantation from the mid to high intertidal zone (Quigley et al. 2020), increased temperatures (Stratil et al. 2013), and after the introduction of axenic cultures (Rao et al. 2006; Longford et al. 2019).

'Ewa Beach is on the southwestern coast of O'ahu, Hawai'i (HI), USA, and is composed of intertidal carbonate benches with abundant macroalgae (Smith 1992). The southwestern coastline of O'ahu is characterised by wet and dry seasons with a relatively consistent air temperature (Cox et al. 2017). Assemblages of macroalgae in the intertidal zones of southwestern O'ahu are diverse, with a high abundance of red and brown algal species, and are shaped by water temperature, wave exposure, and substrate composition (Cox et al. 2017). 'Ewa Beach is within a suburban area with many newly developed recreational sources such as golf courses and beach parks and expanding suburban housing developments. Potentially nutrient‐enriched waters from stormwater input from the surrounding suburban areas were hypothesised to increase the abundance of invasive species in 'Ewa Beach (Lapointe and Bedford 2011). However, the influx of stormwater did not significantly affect the macroalgal assemblages at this site (Cox and Foster 2013). In other locations, runoff may disturb the microbiome of organisms such as sponges (Shore et al. 2021) and those in seawater from a coastal coral lagoon (Meyneng et al. 2024).

In 2019, a snapshot of the microbiota associated with five common macroalgal species at 'Ewa Beach showed that diversity and community composition varied by macroalgal host phylum, followed by thallus complexity, host species, and the host's status as an invasive or native species (Kuba et al. 2021). Our goal was to see if and how these microbiomes varied through time over repeated low‐tide events, which could help predict their response to future climate scenarios. Even though tidal ranges in Hawai'i fluctuate on small scales (< 1 m) (Gosline 1965), algae are exposed for up to 6 h in some locations (Cox and Smith 2015). The spring low tides (maximum low tides of −0.15 m) occur approximately 20 days throughout the year and coincide with warm summer months (Cox and Smith 2015). We hypothesised that the macroalgae's bacterial communities would be altered on short time scales during sequential low tide events due to increases in exposure. All macroalgae were collected in a relatively small area and, therefore, were exposed to the same environmental conditions. Abiotic data were collected in 2023, 2 years after algal sampling, to further characterise the environment at this site, but cannot be directly used in microbiome analysis because of the different sampling years. To examine temporal variation, the microbial communities of seven common macroalgal species from 'Ewa Beach were examined over five consecutive days. Daily collections of macroalgal replicates allowed for the testing of the following hypotheses: (1) changes in bacterial community structure through time will vary depending on macroalgal class and species, (2) bacterial communities of each algal species decrease in diversity and change in structure over the 5‐day sampling period.

## 2 Materials and Methods

### 2.1 Study Site Characterisation and Sample Collection

**FIGURE 1:**
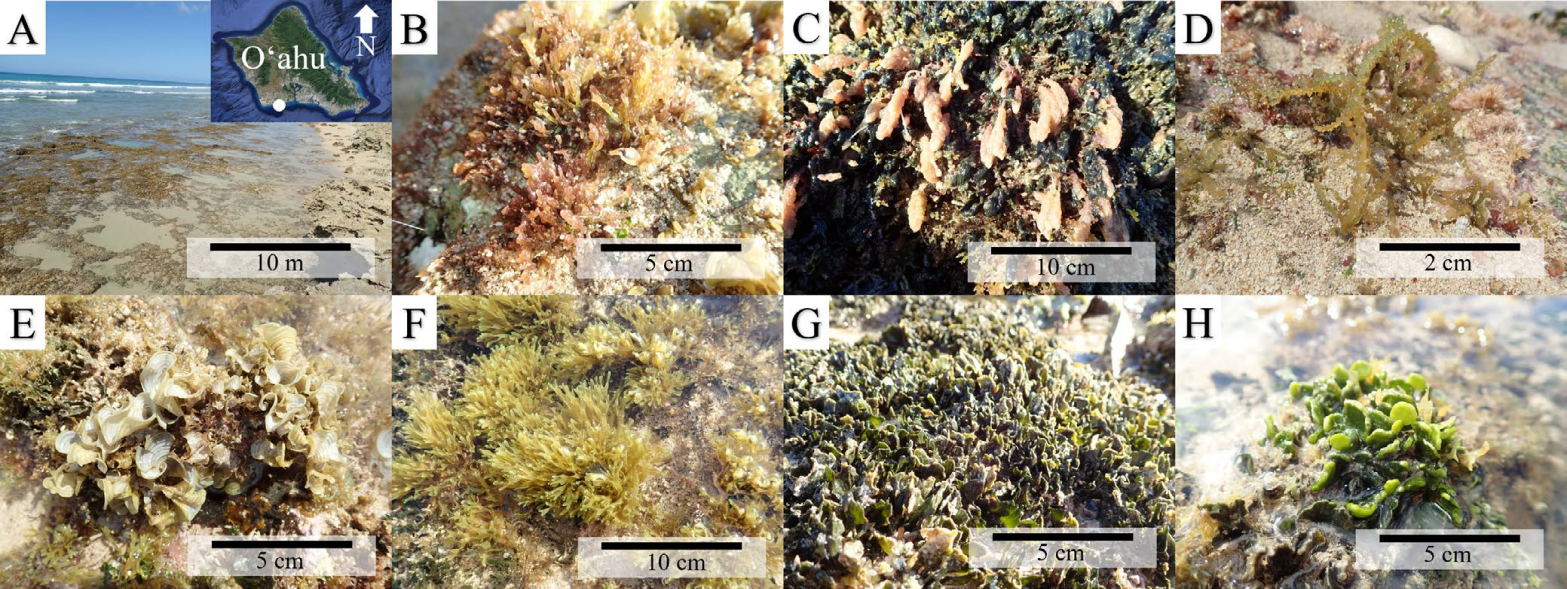
(A) Overview of sampling site at 'Ewa Beach, O'ahu, Hawai'i. Inset of O'ahu from Google Maps. Images of representatives of each algal species collected: (B) *Laurencia dendroidea* (C) *Asparagopsis taxiformis* , (D*) Acanthophora spicifera*, (E) *Padina sanctae‐crucis*, (F) *Dictyota sandvicensis*, (G) *Avrainvillea lacerata* , and (H) *Halimeda discoidea* .

'Ewa Beach is an intertidal bench composed of a mixture of sand and carbonate located on the southwestern coast of O'ahu, Hawai'i (21°18′20.9″ N 158°01′42.2″ W) (Figure 1A). Samples of each targeted algal species were collected in a stratified random design across an approximately 20 × 50 m area parallel to the shoreline for five consecutive days (22–26 May 2021) during low tide events (tidal range = −0.09 to −0.15 m) between 0730 and 0930 when the bench could be safely accessed during its exposure period (Figure 1A). The spatial and temporal variation of macroalgae at this site was described by Cox et al. (2017) [see ‘Site 13’ in Figure 2 of Cox et al. (2017)]. The site was delineated based on GPS and land line‐ups from Cox et al. (2017). Collections were stratified by targeting flat surfaces on the top of the bench and separated by at least 5 m based on the collector's strides. The closest targeted species within reach of the last stride were collected to ensure that samples were distributed across the bench and were not collected from the same algal clump. Time constraints related to safety concerns (i.e., a 30‐min collection window due to the incoming tide on a sharp, rocky intertidal bench), and high wave activity made the deployment of a transect line on the bench with random sampling not possible at this site. Samples (*n* = 3 replicates per species) included the green algae *Avrainvillea lacerata* and *Halimeda discoidea* , red algae *Acanthophora spicifera* , *Asparagopsis taxiformis* , and *Laurencia dendroidea*, and brown algae *Padina sanctae‐crucis* and *Dictyota sandvicensis* (Figure 1). Sample collection methods and the site were identical to those of Kuba et al. (2021). Briefly, samples of individual whole thalli were collected with gloved hands, rinsed with 3.5% sterile artificial seawater, placed in RNAlater, and stored at 4°C overnight before freezing at −80°C. Water samples (50 mL each, *n* = 3) were collected each day as background controls, and replicates from each day were filtered through the same sterile 0.2‐μm PES (polyethersulfone) membrane filter, which was then preserved in 5 mL of RNAlater. Filters were stored at 4°C overnight before freezing at −80°C. Voucher specimens of each species were deposited in the *Herbarium Pacifica*, Bernice Pauahi Bishop Museum, Honolulu, HI (Table S1).

**FIGURE 2:**
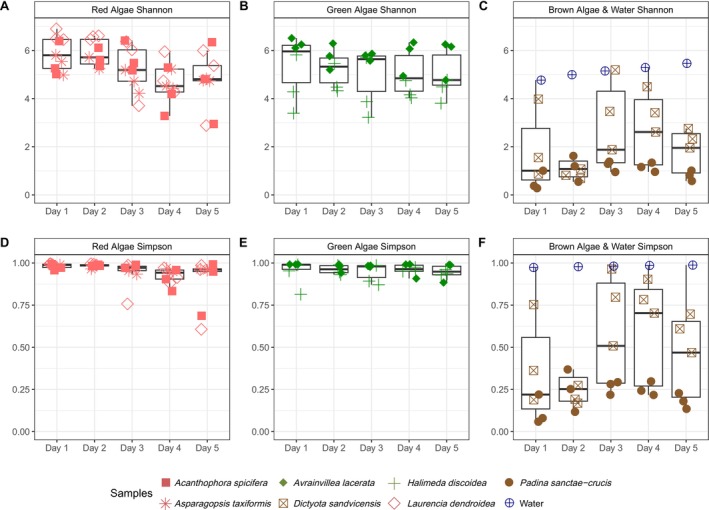
Boxplots of Shannon Diversity for the microbiome of (A) red algae (Florideophyceae), (B) green algae (Ulvophyceae), (C) brown algae (Phaeophyceae) and background water, and Simpson's diversity for the microbiome of (D) red algae (Florideophyceae), (E) green algae (Ulvophyceae), (F) brown algae (Phaeophyceae) and background water collected each day. See Table S3 for significant comparisons.

No abiotic data were collected at the time of sampling due to the aforementioned time constraints. We did, however, collect temperature and light data in 2023, 2 years after algal sampling, solely to qualitatively characterise these conditions at this site; these data were not used in subsequent analyses. Light levels and temperature were recorded over 3 days (15–17 May 2023) during another negative low tide event that had similar weather conditions as the original sampling period. Three HOBO Pendant MX Temperature/Light Data Loggers (Onset Brands, Bourne, Massachusetts, USA) were placed in the high, mid, and low intertidal zones. Light units from the HOBO Loggers were converted from lux to μmol m^−2^ s^−1^ by converting lux to klux (1000 lx = 1 klux) and multiplying by a conversion factor of 18 for sunlight per the LI‐COR protocol (Biggs 2022). Sensors were only deployed for 3 days in 2023 due to inclement weather conditions. Average temperature was between 25.89°C ± 0.35°C and 26.57°C ± 0.74°C, and average light ranged from 325.54 ± 111.79 μmol m^−2^ s^−1^ to 456.40 ± 280.83 μmol m^−2^ s^−1^ (Table S2). These data and results are to be used only to gain an understanding of abiotic variability within this intertidal bench during low tide and cannot be used in the interpretation of the 2021 sampling dataset.

### 2.2 DNA Extraction, Library Preparation, and Sequencing

DNA extraction was performed as previously described using the MP Biomedicals FastDNA Spin Kit manufacturer's protocol (Kuba et al. 2021). Briefly, 0.5 g of whole algal thallus was weighed using sterile techniques and 70% ethanol flame‐sterilised forceps. Samples weighing more than 0.5 g were split into two tubes for bead beating and pooled following extraction. DNA was then quantified using a Qubit 4.0 fluorometer (Invitrogen, Carlsbad, California, USA) and Qubit HS dsDNA Quantification Assay Kit (ThermoFisher Scientific, Waltham, Massachusetts, USA), following the manufacturer's protocol. The DNA of seawater background controls was pooled by collection day prior to library preparation due to low DNA concentrations following extraction.

PCR was performed using primers 340F (5′‐CCTACGGGNGGCWGCAG‐3′) and 784R (5′‐GGACTACHVGGGTATCTAATCC‐3′; Klindworth et al. 2013) that target variable regions 3 and 4 of the bacterial 16S ribosomal RNA (rRNA) gene and included Illumina overhang sequences used in the subsequent indexing PCR. PCR was performed in triplicate following a previously published protocol (Kuba et al. 2021). PCR products were visualised on a 2% agarose gel pre‐stained with GelRed (Biotium, Fremont, California, USA) under UV light. Triplicate reactions were pooled, cleaned with AMPure XP beads (Beckman Coulter, Brea, California, USA) following the manufacturer's protocol, and quantified with a Qubit 4.0 fluorometer as above. Cleaned amplicons were sent to North Carolina State University's Genomics Core (Raleigh, North Carolina, USA) for indexing and sequencing on an Illumina MiSeq using v3 chemistry (2x300 bp) following the manufacturer's protocol.

## 3 Data Analysis

Demultiplexed sequences were assessed for quality using FastQC (Andrews 2010). Forward and reverse primers were trimmed using cutadapt (Martin 2011). Quality filtering, error correction, chimaera detection and filtering, and identification of amplicon sequence variants (ASVs) was done using the Divisive Amplicon Denoising Algorithm (DADA2) package v.1.26.0 (Callahan et al. 2016) as implemented in R 4.1.2 (R Development Core Team 2010) following a previously described protocol (Kuba et al. 2021). SILVA v.138.1 database was used for taxonomic assignment (Quast et al. 2013) via the IdTaxa function in the DECIPHER package version 3.2.0 (Murali et al. 2018). Any ASVs identified as chloroplast 16S rRNA gene sequences were removed prior to downstream analyses.

Statistical analyses were performed in R software 4.0.1 (R Core Team 2025) using phyloseq v.1.38.0 (McMurdie and Holmes 2013), vegan v.2.6–6.1 (Oksanen et al. 2022), and microbiome v.1.23.1 (Lahti and Shetty 2017). Visualisations for relative abundance, alpha diversity metrics, and dissimilarity over time were made with ggplot2 v.3.5.1 (Wickham 2016). Alpha diversity metrics were examined across time for each macroalgal species with an ANOVA (analysis of variance) for each species diversity metric, followed by a Tukey's HSD post hoc test to compare day‐to‐day differences. The ANOVA assumptions of homogeneity of variance and normality were tested with a Bartlett's test and Shapiro–Wilk normality test, respectively, via the stats package v.3.6.2 (R Core Team 2025). When ANOVA assumptions were not met, a Kruskal‐Wallis test was performed, followed by Dunn's post hoc test with a Bonferroni‐Holm correction. Beta diversity was visualised using non‐metric multidimensional scaling (NMDS) ordination plots with ellipses representing 95% confidence intervals using proportional transformations based on Bray–Curtis dissimilarity via ggordiplots v.0.4.3 (Quensen et al. 2024). Differences in beta diversity amongst macroalgal classes (average of all times) and through the sampling period were tested using permutational analysis of variance (PERMANOVA), which were preceded by PERMDISP2 to test for multivariate homogeneity of group dispersions in vegan (Oksanen et al. 2022). Bray–Curtis dissimilarity of all samples of each macroalgal species was plotted by collection day (Euclidean distance matrix of sample days) using vegan to calculate matrices (Oksanen et al. 2022) and was analysed with a regression analysis. For macroalgal species that had significant changes in their community structure through time, we identified specific ASVs contributing to these differences using similarity percentage analysis (SIMPER) for each individual macroalgal species on ASVs greater than 0.1% relative abundance in vegan (Oksanen et al. 2022). The top five ASVs identified by SIMPER contributing to day‐to‐day dissimilarities for each day comparison through the sampling week were chosen to be investigated further because these ASVs had high contributions to temporal differences; contributions to day‐to‐day dissimilarity were low (< 1%) after these top 5 ASVs. We classified core ASVs as those present in all three macroalgal replicates per species on each day of collection to include those present in all samples on each day for each macroalgal species in order to avoid inclusion of transient ASVs. Venn diagrams comparing the number of core ASVs present on individual collection days and those present on all days were generated using VennDiagram v.1.7.3 (Chen and Boutros 2011).

## 4 Results

### 4.1 Sample Collection

The seven most abundant macroalgal species were collected and identified morphologically using (Abbott 1999 and Abbott and Huisman 2004). Collected algae exhibited a diversity of thallus morphologies, including upright axes with either rounded or filamentous tips, fan‐shaped blades, and flattened segments (Figure 1). Additionally, *H. discoidea* and *P. sanctae‐crucis* were calcified (Figure 1). Most of these species are native to Hawai'i except for *Av. lacerata* and *Ac. spicifera*, which are invasive and have been distributed throughout the Main Hawaiian Islands since their introduction in 1981 and 1952, respectively (Doty 1961; Brostoff 1989) (Figure 1). All macroalgal specimens were found inhabiting the same space in the intertidal bench, often growing in very close proximity (Figure 1), except for *Ac. spicifera* specimens, which were found in the high intertidal, and *L. dendroidea*, which was often found higher vertically (i.e., growing epiphytically on top of other species, most commonly *Av. lacerata*).

### 4.2 Sequencing Analysis

The microbiomes from a total of 105 macroalgal samples and 5 background seawater controls were sequenced, resulting in 7,629,545 reads (average 69,360 reads per sample) with an average length of 414 ± 15 base pairs after quality control and filtering. A total of 30,490 ASVs remained after the assembly of paired reads and removal of chimaeras and chloroplast 16S rRNA gene sequences. The average number of observed bacterial ASVs was 1598.39 ± 706.70 for all samples, ranging from 329 (a specimen of *P. sanctae‐crucis* on day one) to 3204 (a specimen of *Ac. spicifera* on day five; Table S3). Rarefaction curves based on the number of observed ASVs reached a plateau, indicating sufficient community representation of each macroalgal sample (Figure S1).

### 4.3 Alpha and Beta Bacterial Diversity

Averaged across the five sampling days, the number of observed ASVs, Shannon, and Simpson's diversity indices significantly varied by macroalgal class and significantly differed from those of the background water controls (Tables S4 and S5). Pairwise comparisons showed that brown algae were associated with significantly fewer and less diverse bacterial taxa compared to those associated with the red and green algae (Table S5). Bacterial diversity associated with brown macroalgae was also significantly lower than that of background water controls (Kruskal‐Wallis *p*‐value < 0.05; Table S5). The environmental water samples (controls) had fewer observed ASVs compared to those associated with the red and green macroalgae (Observed ASVs: ANOVA *p*‐value < 0.05; Table S5).

For *L*. *dendroidea*‐associated bacteria, diversity significantly decreased over time (Kruskal‐Wallis *p*‐value < 0.05, Table S5). Whilst post hoc pairwise comparisons between days were not significant (Table S5), diversity slightly decreased over time (Figure 2). Bacteria of *As. taxiformis* showed significant variations in diversity (ANOVA *p*‐value < 0.05) (Figure 2, Table S5); only Simpson's diversity was lower on day one and only compared to day three (Simpson's: ANOVA *p*‐value < 0.05, *F* = 4.82, df = 4, Table S5, Figure 2). *Ac. spicifera*‐associated bacteria had significantly lower Simpson's diversity on day two compared to day four (Kruskal‐Wallis *p*‐value < 0.05, *χ* ^2^ = 5.43, df = 4, Figure 2, Table S5). Simpson's diversity for *D. sandvicensis*‐associated bacteria was significantly lower on day two compared to days three and four (ANOVA, *p*‐value < 0.05, *F* = 5.34, df = 4, Figure 2, Table S5). Bacteria associated with *P. sanctae‐crucis* and both green macroalgae (*Av. lacerata* and *H. discoidea* ) did not vary significantly in richness or diversity over time.

**FIGURE 3:**
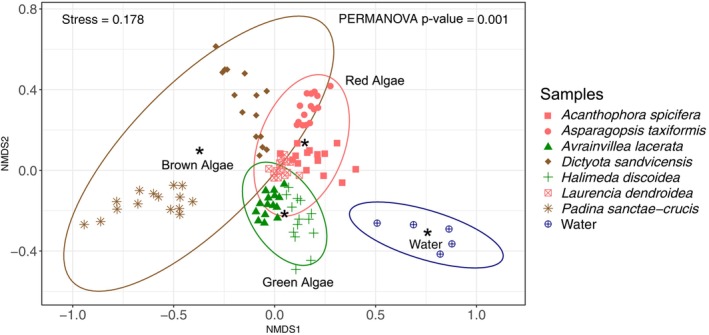
Non‐metric multidimensional scaling (NMDS) ordination based on Bray‐Curtis dissimilarities showing clustering of individual samples and their bacterial community composition based upon macroalgal class. Labels and asterisks (*) indicate group centroids. Ellipses represent 95% confidence intervals. See Table S3 for PERMANOVA results grouped by macroalgal class.

The NMDS plot based on Bray‐Curtis dissimilarities of all specimens from all collection days showed distinct bacterial communities amongst the macroalgal classes (PERMANOVA, *p*‐value < 0.05, PERMDISP2, *p*‐value = 0.39, Tables 1 and S6), and all were distinct from those found in the environmental water (control) samples (PERMANOVA, *p*‐value < 0.05, Figure 3). Bacteria associated with the brown algae were the most distinct, with those of *P. sanctae‐crucis* being the most different amongst all species collected (Figure 3). Within the red algae, *As*. *taxiformis* had the most distinct microbiome, whilst the bacterial communities of *L. dendroidea* and *Ac. spicifera* somewhat overlapped (Figure 3).

**TABLE 1:** Summary of PERMANOVA (permutational analysis of variance) and PERMDISP2 (permutation test of multivariate homogeneity of groups dispersion) results based on Bray‐Curtis dissimilarities of macroalgal‐associated bacterial amplicon sequence variants amongst groups.

Macroalgal class	Dataset	Factor	PERMANOVA *p*	PERMDISP2 *p*
All	All	Macroalgal class	**0.001** *	0.393
		Day	0.930	0.947
Florideophyceae	*Acanthophora spicifera*	Day	**0.001** *	0.435
	*Asparagopsis taxiformis*	Day	0.076	0.976
	*Laurencia dendroidea*	Day	**0.026** *	0.944
Ulvophyceae	*Halimeda discoidea*	Day	0.250	0.878
	*Avrainvillea lacerata*	Day	0.233	0.890
Phaeophyceae	*Dictyota sandvicensis*	Day	**0.017** *	0.263
	*Padina sanctae‐crucis*	Day	0.157	0.426

*Note:* Significance values are in bold. < 0.05.

* *p* < 0.05.

**FIGURE 4:**
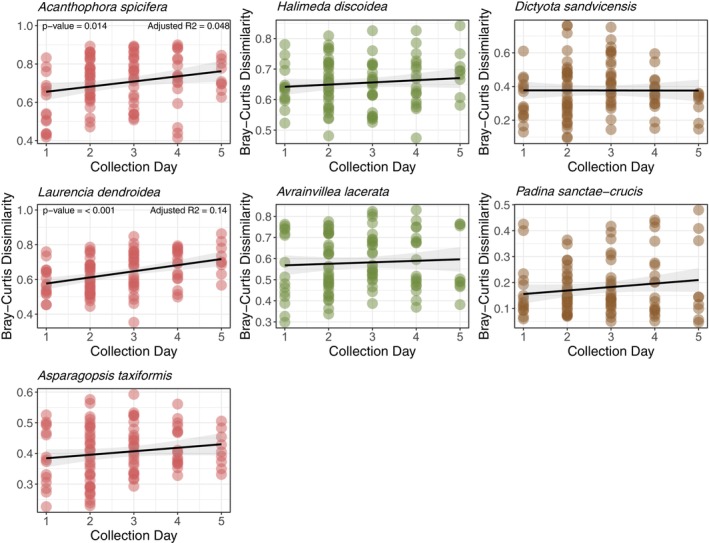
Scatter plots of temporal distance (Euclidean distance matrix of sampling days) and community dissimilarity (Bray‐Curtis distance) for all macroalgal samples.

Of the seven species of macroalgae, only three significantly varied in their bacterial community structure through time: both red algae (*Ac. spicifera*, *L. dendroidea)* and the phaeophycean *D. sandvicensis* (PERMANOVA, *p*‐values < 0.05, PERMDISP2, *p*‐values > 0.05, Tables 1 and S6). Bray‐Curtis bacterial community dissimilarity for both red algae significantly increased through time, but adjusted *R* ^2^ values were low (*Ac. spicifera*: adjusted *R* ^2^ = 0.0479, *p* < 0.05; *L. dendroidea*: adjusted *R* ^2^ = 0.1378, *p* < 0.05; Figure 4), and there are likely other, unmeasured drivers of change over time. In contrast, the microbiomes of the green algae *Av. lacerata* and *H. discoidea* , red alga *As. taxiformis*, and brown alga *P. sanctae‐crucis* exhibited no significant differences in community structure over time (Tables 1 and S6).

**FIGURE 5:**
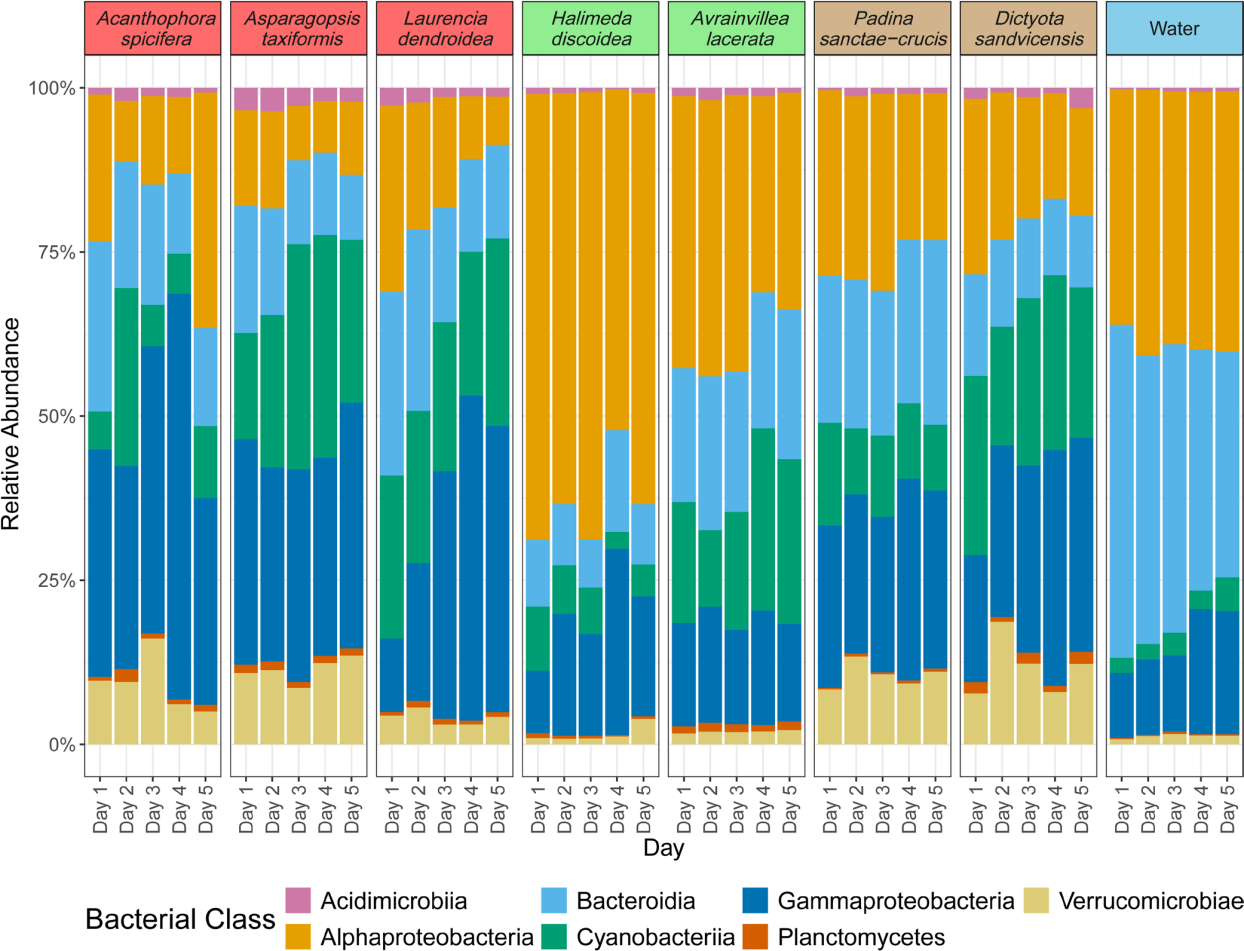
Average relative abundance of the top 5% most abundant bacterial classes for all macroalgal species and background water collected each day. Highlighted species name corresponds to macroalgal class. Red = Florideophyceae, green = Ulvophyceae, brown = Phaeophyceae, and blue = seawater.

Bacterial ASVs were differentially distributed amongst the different macroalgal classes and species based on the average relative abundances. Bacterial class relative abundances varied over the five consecutive low tides, with more variation observed in those associated with the red algae *Ac. spicifera* and *L. dendroidea* (Figure 5). At the class level, Alphaproteobacteria, Gammaproteobacteria, Cyanobacteriia, and Bacteroidia ASVs were the most relatively abundant for all macroalgal species (Figure 5). Green macroalgae had higher relative abundances of Alphaproteobacteria ASVs and a lower abundance of Verrucomicrobiae ASVs compared to other species, with *H. discoidea* having the highest relative abundance of Alphaproteobacteria ASVs of all species (Figure 5).

Temporally, changes in average relative abundance were variable over the 5 days for different macroalgal species. For *Ac*. *spicifera*, associated Alphaproteobacteria ASVs decreased in relative abundance from 22.32% on day one to 9.26% on day two, with similar relative abundances on days three and four, but increased to 35.85% on day five; the Cyanobacteriia ASVs increased from 5.76% on day one to 27.13% on day two and then decreased with a slight increase again on day five (11%). *L*. *dendroidea*‐associated Gammaproteobacteria ASVs clearly increased (from 11.17% to 49.53%) whilst Alphaproteobacteria ASVs decreased (from 28.37% to 7.35%) in relative abundance over time (Figure 5). The microbiome associated with *As. taxiformis* was more stable over time, with only a slight increase in the relative abundance of Cyanobacteriia ASVs (Figure 5). Additionally, we saw less temporal variation in the relative abundance of bacterial taxa (e.g., Verrucomicrobiae ASVs and both Alpha*‐* and Gammaproteobacteria sequences) associated with *D. sandvicensis* compared to that seen in association with the other brown alga, *P. sanctae‐crucis* (Figure 5). Background water controls were stable over the 5 day sampling period and had a high abundance of Alphaproteobacteria, Bacteroidia, and Gammaproteobacteria ASVs (Figure 5).

### 4.4 ASVs Associated With Temporal Change

**FIGURE 6:**
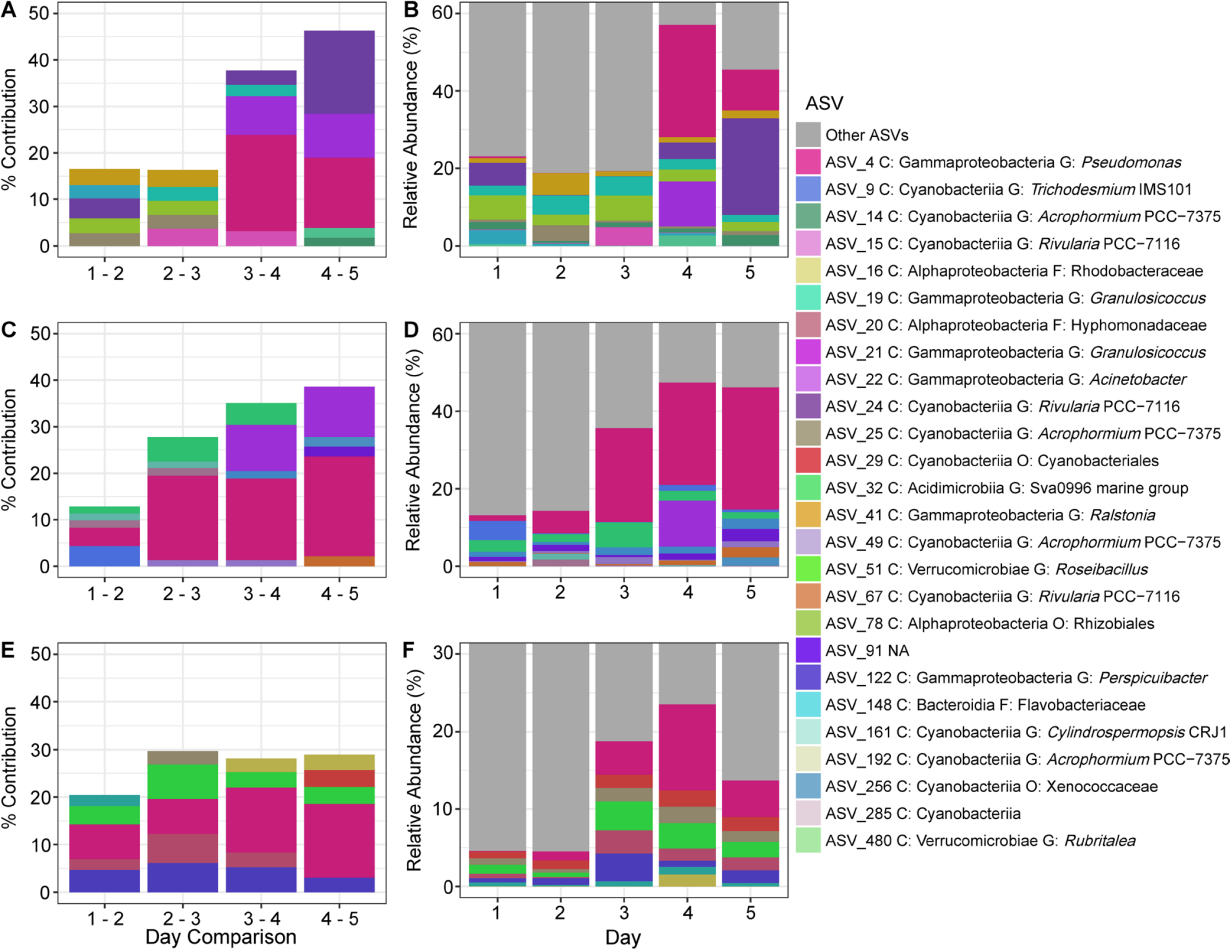
Relative abundance and percent contribution to day‐to‐day dissimilarity of the top five ASVs (amplicon sequence variants) identified by SIMPER analysis for (A, B) *Acanthophora spicifera* , (C, D) *Laurencia dendroidea*, and (E, F) *Dictyota sandvicensis*. The left panel (A, C, E) shows the percent contributions of individual ASVs to Bray–Curtis dissimilarity between consecutive sampling days as calculated by SIMPER. The right panel (B, D, F) shows the relative abundance of the ASVs identified by SIMPER across the sampling days. Only the top five ASVs contributing the most to day‐to‐day dissimilarity are shown for each species, and colours correspond to ASV, with grey representing the rest of the community not significantly contributing to dissimilarity. Colours across panels and species are consistent to help track individual ASVs patterns.

SIMPER identified 26 ASVs as high contributors (the top five ASVs contributing to dissimilarity) to microbial dissimilarity across days for *Ac. spicifera, L. dendroidea*, and *D. sandvicensis*, with each macroalgal species analysed individually (Figure 6). In all three macroalgal species, the ASVs identified by SIMPER increased in relative over the five sampling days (Figure 6). Of these 26 ASVs, 18 were identified to genus level, three at both family and order level, one at class level, and one ASV could not be classified (Figure 6). The nearest BLAST result of the unclassified ASV was to a sequence of *Clostridium aestuarii* , but with only 76% similarity (Table S7). BLAST results of the 26 ASVs identified differed from the SILVA v.138.1 database results at lower taxonomic levels for some ASVs, but were overall consistent with the SILVA database (Table S7). ASVs assigned to the classes Cyanobacteriia (12) and Gammaproteobacteria (6) contributed most to the day‐to‐day temporal bacterial dissimilarity associated with *Ac*. *spicifera*, *L. dendroidea*, and *D. sandvicensis* (Figure 6). ASV 4 (*Pseudomonas* sp.) was present on all collection days and contributed highly to dissimilarity for *Ac*. *spicifera*, *L. dendroidea*, and *D. sandvicensis* amongst collection days (Figure 6). The number of core ASVs (those present for a species in all triplicate samples collected each day) varied amongst macroalgal species, ranging from 334 ASVs in *As. taxiformis* to 42 ASVs in *P. sanctae‐crucis* (Figure S2). Additionally, differences were seen in the amount of core ASVs present on individual days amongst species (Figure S2). For example, *D. sandvicensis* had core ASV numbers ranged from three to 130 ASVs present on days two and four respectively, whilst *As. taxiformis* had less variation, ranging from 37 to 88 ASVs on days five and one respectively (Figure S2).

For *Ac. spicifera*, 11 ASVs were identified and cumulatively contributed 16.34% to 46.31% to the dissimilarity for each daily comparison (Figure 6A). These 11 ASVs ranged in relative abundance from 18.75% on day two to 56.25% on day four of the total community composition for each day (Figure 6B). Five of the 11 ASVs associated with *Ac. spicifera* were identified to genus‐level: *Granulosicoccus* (two ASVs), *Pseudomonas* (one ASV), *Acinetobacter* (one ASV), *Acrophormium* PCC‐7375 (two ASVs), *Rubritalea* (one ASV); two to family level: one as Rhodobacteraceae and one as Flavobacteriaceae; one to order level: Hyphomicrobiales, and lastly the one that could not be classified mentioned above (Figure 6A,B). ASV 4, identified as *Pseudomonas*, had high relative abundances on days four and five; whereas the Rhodobacteraceae ASV was present on day one and then was not present in high abundance again until days four and five (Figure 6B). Other ASVs such as those identified as *Granulosicoccus, Acrophormium* PCC‐7375, and Hyphomicrobiales were present throughout the sampling period in varying relative abundances. The *Acinetobacter* ASV was only detected on day four, and the unidentified ASV only on day three (Figure 6B).

As with *Ac. spicifera*, 11 ASVs contributed to the dissimilarity of *L. dendroidea* between‐day comparisons, ranging from 12.83% to 38.62% of the total dissimilarity cumulatively (Figure 6C). The total relative abundance of these ASVs in *L. dendroidea* increased over the sampling period, from 12.5% to 50% of the total community (Figure 6D). Influential ASVs for *L. dendroidea* consisted of *Trichodesmium* IMS101 (one ASV), *Rivularia* PCC‐7116 (three ASVs), *Cylindrospermopsis* CRJ1 (one ASV), *Pseudomonas* (one ASV), *Ralsonia* (one ASV), and *Acinetobacter* (one ASV) (Figure 6C,D). The abundance of *Pseudomonas* increased greatly in *L. dendroidea* over the five sampling days, whilst the abundance of other ASVs varied over time. For instance, *Trichodesmium* IMS101 decreased in abundance after day one, whilst *Rivularia* PCC*‐*7117 fluctuated throughout the sampling period. *Acinetobacter* was in high abundance on day four but was not present on other days (Figure 6D).

Eight ASVs contributed to the dissimilarity of the microbiome of *D. sandvicensis* amongst days, ranging from 20.42% to 29.69% of the total dissimilarity cumulatively (Figure 6E). The proportion of the bacterial community composed of these ASVs increased over the five sampling days, ranging from 4% of the community at the lowest abundance on day one to 24% at the highest abundance on day four (Figure 6F). The influential ASVs contributing to differences amongst days for *D. sandvicensis* consisted of *Acrophormium* PCC‐7375 (three ASVs), *Pseudomonas* (one ASV), *Perspicuibacter* (one ASV), *Roseobacillus* (one ASV), Sva0996 marine group (one ASV), and the family Hyphomonadaceae (one ASV) (Figure 6E,F). The ASVs identified as genera *Pseudomonas* and Sva0996 marine group had the largest increases in relative abundance over the sampling period, whilst ASVs of other genera varied in abundance over the sampling period (Figure 6F). ASV 122, identified as *Perspicuibacter*, had a notable decrease in relative abundance between days one and two, and the three ASVs identified as *Acrophormium* PCC‐7375 were variably abundant throughout the sampling period (Figure 6F). The relative abundance of *Roseobacillus* (ASV 51) was also variable over time, being the most abundant on day three (Figure 6F).

## 5 Discussion

Diversity and community structure of the macroalgal associated‐bacterial communities varied over time for only three species, whilst the bacterial community structure of four other species did not change over the five‐day collection period. Estimated bacterial abundance has been seen to vary over time differently depending on algal host species before, but on longer timescales throughout a 12 month period (Brunet et al. 2025). Other shorter term changes in bacterial communities associated with the red alga *Delisea pulchra* have been observed just 15 days after being transplanted to an aquarium environment (Zozaya‐Valdés et al. 2016). As expected, bacterial communities were distinctive amongst macroalgal classes, and the microbial community composition was similar to those communities found associated with macroalgae at ‘Ewa’ and in the Hawaiian Archipelago collected 2 years prior: Alphaproteobacteria, Bacteroidia, Cyanobacteriia, Gammaproteobacteria, and Verrucomicrobiae were highly abundant in all (Kuba et al. 2021, 2023). Alpha diversity of brown algae was lower than that of red and green across all metrics, with differences in microbial diversity aligning with previous studies (Kuba et al. 2021, 2023; Chen et al. 2022; Nahor et al. 2024) and further supporting microbial variation by taxonomic classification. Macroalgal species have been shown to produce a variety of secondary metabolites and compounds that have antimicrobial properties (Pérez et al. 2016), which may influence colonising bacteria, leading to differences in alpha diversity. Some variables (e.g., invasive or native status, calcification, and thallus morphology) could not be reliably tested due to confounding factors amongst macroalgal species (i.e., different species have different thallus morphologies, calcification levels, or invasive/native status, which limited our ability to test if differences were due to these variables or host species specificity).

Differences observed in the bacterial community of some macroalgal species over time may be attributed to variations in environmental conditions during consecutive low tides during the five‐day sampling period. Abiotic stressors such as desiccation often impact algal assemblages in upper intertidal zones (e.g., Schonbeck and Norton 1979). The microbial communities of the kelp *Ecklonia radiata*, when thermally stressed and experiencing thallus bleaching, were distinct from those of healthy hosts (Marzinelli et al. 2015); thermal stress has been shown to alter the microbial communities of macroalgal hosts (Morrissey et al. 2021; Castro et al. 2024; Vadillo Gonzalez et al. 2024). Desiccation, along with short‐term changes in temperature, light, and salinity during tidal transitions, may also increase the release of dissolved organic carbon in macroalgae, potentially altering the associated microbial communities (Wyatt et al. 2014; Zhao et al. 2023; Bennett et al. 2024). Fluctuations in temperatures and light intensity during tidal cycles could impact microbial communities, especially when repeated over consecutive days. Large fluctuations in light intensity were seen at this site in 2023, but for reliable conclusions to be drawn, data would need to be collected at the same time as macroalgal sampling. Additionally, macroalgae at 'Ewa Beach were exposed for a short amount of time compared to other intertidal areas (e.g., temperate regions with larger tidal ranges), meaning they were not fully desiccated (i.e., still damp) at the time of collection. Differences in the thallus morphology amongst macroalgal species (e.g., thin individual thalli in *D. sandvicensis* versus the fan‐shaped thalli of *P. sanctae‐crucis* that curls in on itself) and clumping on the substrate could increase the retention of moisture within thalli during low tides, allowing them to have a more stable microbial community. The number of core ASVs differed amongst species, and varied over the 5‐day sampling period for each species, showing potential variations in the core microbiota of species such as *D. sandvicensis*. Because we used a strict definition of core ASVs (ASVs with 100% occurrence in all triplicate samples per day) to avoid inclusion of transient ASVs and remain consistent with Kuba et al. (2021), it is possible that we missed some potentially important taxa (Neu et al. 2021). Further research is needed to investigate these potential abiotic drivers, morphological differences, and their impact on macroalgal microbial communities.

Bacterial ASVs contributing to temporal change over the five sampling days (as determined by SIMPER) all belonged to the classes identified as the top 5% most relatively abundant, suggesting they could play a foundational role in the microbial community of these macroalgae. However, these ASVs were identified via SIMPER, which does not identify ecological function, only those ASVs contributing to day‐to‐day dissimilarities. ASVs of *Pseudomonas* were in relative abundance through time for all three macroalgal species whose bacterial communities varied. *Pseudomonas* species, however, are diverse and can benefit or harm their macroalgal hosts. Species such as *P. aeruginosa* produce antimicrobial compounds (Goecke et al. 2010; Nagel et al. 2012), induce morphogenesis (Nakanishi et al. 1996), and may help promote growth as observed in other organisms via nitrogen fixation (Khumairah et al. 2022) and alleviate drought stress in plant species (Papadopoulou et al. 2022). Other species of *Pseudomonas* sp. can cause disease, such as green spot rotting in *Pyropia yezoensis* (Nakao et al. 1972). Unfortunately, the 16S rRNA gene region used for most bacterial metabarcoding studies cannot differentiate species, so additional work is needed to identify those species present at 'Ewa Beach. *Acinetobacter* has been observed to increase in abundance over time on decaying freshwater macroalgae within 6 h (Chun et al. 2017). In marine environments, *Acinetobacter junii* PS12B has been isolated from seaweeds, seawater, and sediments from the east coast of India, and has been shown to produce agarase and degrade the red macroalgal polysaccharide carrageenan (Leema Roseline and Sachindra 2016). The peak in *Acinetobacter* ASVs that we saw, therefore, could be an indicator of host health, however, data from metagenomics and/or functional assays of isolated bacterial species would need to be done to further support this hypothesis. Lastly, ASVs of *Acrophormium* PCC‐7375 contributed to the differences observed in the red algae *Ac*. *spicifera* and *L*. *dendroidea* bacterial communities through time and was one of the most abundant bacteria in association with the red algae at 'Ewa Beach in the Kuba et al. (2021) study. Again, additional work is necessary to identify species and understand their functional role in the health of their hosts.

Some ASVs that contributed to the temporal dissimilarity of *Ac. spicifera*‐associated bacteria were those of taxa that could support its health. Members of Rhodobacteraceae may synthesise vitamin B_12_ and help protect the macroalgal host (Croft et al. 2005; Dogs et al. 2017). Rhodobacteraceae abundance has also been positively correlated with intertidal macroalgal stress levels in the high intertidal zone, potentially indicative of variable stress levels over time due to their higher abundance later in the sampling period and potentially higher thermal tolerance (Quigley et al. 2020). Samples of *Ac. spicifera* at 'Ewa Beach were most commonly found in the high intertidal zone, so future studies should consider whether species of Rhodobacteraceae are helping mitigate the stress experienced in this intertidal position and directly measure host stress. Flavobacteriaceae ASVs were highest in relative abundance on day one, and whilst these taxa can be associated with benefits to the host macroalga via protective compounds (Zan et al. 2019), they also have been found to be enriched on hosts experiencing bleaching disease (Zozaya‐Valdes et al. 2015) and can degrade whole algal tissues (Brunet, de Bettignies, et al. 2021; Barbeyron et al. 2023). Other taxa may have increased due to chemical changes in the host in response to stress (i.e., 5 days of repeated low tide exposure). For instance, stressed algae release more halogenated compounds (Laturnus et al. 2000), which some Hyphomicrobiales taxa can metabolise (Lavecchia et al. 2024), and thus may lead to the higher relative abundance of Hyphomicrobiales ASVs that we saw on the last day of sampling. Species of *Rubritalea* (Verrucomicrobiales) produce squalene (Yoon et al. 2007, 2008), which is a precursor to steroids and D vitamins (Bloch 1983) and support the growth of some macroalgal species (Fries 1984). The ASV identified as *Rubritalea* was only found in high abundance on day four, indicating that whilst it may have benefits for the host alga, it may be transient in the microbiota, and not a member of the core microbiome. Species of *Granulosicoccus* possess several carbohydrate‐active enzymes, and are involved in nitrogen and sulphur cycling, and vitamin B_12_ synthesis (Kang et al. 2018; Weigel et al. 2022). Decreases in this beneficial bacterium at the end of our sampling period during low tides may impact the health of *Ac. spicifera* as it is exposed to repeated stressful environmental conditions.

Temporal differences in *L. dendroidea* were primarily due to increases and decreases in the relative abundance of Cyanobacteriia. The Cyanobacteriia associated with *L. dendroidea* may supply the alga with fixed nitrogen (de Oliveira et al. 2012). Some cyanobacterial ASVs contributing to differences amongst days include nitrogen fixers. *Trichodesmium* is a well‐documented nitrogen‐fixing bacterium, often found in tropical oligotrophic waters (Capone et al. 1997), and *Rivularia* was found in high abundance in macroalgae at 'Ewa Beach but was more abundant in association with brown and green macroalgae (Livingstone et al. 1984; Kuba et al. 2021). *Cylindrospermopsis* is a Cyanobacteriia that fixes atmospheric nitrogen under limited nitrogen conditions and produces the cyanotoxin cylindrospermopsin (Walsh et al. 2009; Willis et al. 2016). Whilst *Cylindrospermopsis* is common in freshwater, it has the capacity to tolerate higher salinity environments under enriched nitrogen conditions (Calandrino and Paerl 2011), making its presence potentially an indicator of high nitrogen levels. The presence and abundance of these Cyanobacteria may vary in response to increased nutrient inputs, either anthropogenic or natural. One hypothesis is that stormwater input from the suburban developments and golf courses near this site may act as an input of excess nutrients; however, such inputs have not been observed to alter 'Ewa Beach algal assemblages themselves (Cox and Foster 2013). Nearshore submarine groundwater discharge may be another source of increased nutrients and has been observed in other intertidal and shallow subtidal sites in Hawaii (Dailer et al. 2010; Amato et al. 2018). Targeted sampling of nutrient sources and host–microbe interactions are needed to understand their influence on bacterial community composition in the future and their role as potential nitrogen sources. *Ralstonia* ASVs varied in relative abundance throughout the sampling period, and these bacteria can compromise host health through interactions with natural macroalgal microbiota (Li et al. 2022). It should be noted that *L*. *dendroidea* was commonly found growing epiphytically atop other species (most commonly *Av. lacerata*) and thus occurred higher vertically. This may have left individual *L*. *dendroidea* exposed for longer periods of time and influenced changes in the associated microbial communities. Additionally, its proximity to other macroalgal species may have influenced its associated bacteria.

Members of Hyphomonadaceae may potentially increase in abundance in warmer seawater temperatures (Comba González et al. 2021) and can denitrify and utilise nitrate, potentially making them important to the processes of nitrogen cycling (Weiner et al. 1985). Additionally, members of the Hyphomonadaceae have been seen to induce normal morphogenesis in protoplasts of the red alga *Pyropia yezoensis* (Fukui et al. 2014). ASVs of Sva0996 marine group (class Acidimicrobiia) were a large contributor to daily microbial changes in *D. sandvicensis* with variable relative abundance over the 5 days. Taxa within the Sva0996 marine group act as secondary colonisers on the surface of degrading brown macroalgae and may utilise dissolved compounds and contribute to the nitrogen cycle (Orsi et al. 2016; Brunet, Le Duff, et al. 2021). Association of Sva0096 ASVs with *D. sandvicensis* throughout the sampling period is likely due to soluble compounds being exuded by the macroalgal host, similar to the increase in Hyphomicrobiales seen on the last day of sampling for *Ac. spicifera*. *Roseobacillus* (class Verrucomicrobiae) ASVs were observed in high abundance only on day three. Whilst little is known about bacteria in this genus, Verrucomicrobiae possess antimicrobial secondary metabolites, reducing biofouling on the surface of other brown macroalgae, and serve as indicators of stress as they appear to decrease in abundance with increased host stress (Vollmers et al. 2017; James et al. 2020). Lastly, *Perspicuibacter* (class Gammaproteobacteria) ASVs decreased in abundance between days one and two in *D. sandvicensis* and have been observed as a dominant genus found on tropical coral reefs in good health (Titioatchasai et al. 2023; Yang et al. 2023).

## 6 Conclusions

The microbial communities associated with *Ac. spicifera*, *L. dendroidea*, and *D. sandvicensis* varied whilst those of *As*. *taxiformis*, *H. discoidea* , *Av. lacerata*, and *P. sanctae‐crucis* were stable over the 5‐day sampling period. Numerous ASVs of different bacterial taxa contributed to these changes over time, with many showing indications of being potentially beneficial to host health. However, whether these changes in microbial community were due to variations in abiotic conditions could not be assessed with this data set due to a lack of concurrent abiotic data and a short sampling period. To determine if these changes were caused by fluctuations in abiotic conditions experienced at low tide, future studies should include experimental manipulation of factors such as temperature and solar irradiance to determine if these are important drivers of bacterial community composition change. Sampling periods longer than 1 week may capture cycles of bacterial abundance and species composition that may change or recover over longer tidal cycles and time scales. Coupled sampling of microbiome samples, stable isotope data, and metagenomics may help determine if changes in bacteria associated with nitrogen cycling could be impacted by the surrounding development and anthropogenic input. Lastly, more studies are needed to delineate the functional roles of these bacteria in macroalgal microbial communities. Many of the current experimental studies on macroalgal microbiomes have focused on altering abiotic conditions in laboratory settings, but in situ manipulations of the microbiota present on macroalgae are needed to delineate the functional roles of bacteria (Marzinelli et al. 2024). Understanding the functions and changes of these microbial communities will help to predict how these communities may respond to future climate change scenarios, where temperatures and extreme events such as marine heatwaves are predicted to increase in intensity and frequency.

## Author Contributions

**Evan S. Dunn:** investigation, writing – original draft, visualisation, writing – review and editing, formal analysis. **Heather L. Spalding:** conceptualization, investigation, funding acquisition, methodology, writing – review and editing, validation, formal analysis, data curation, supervision, resources. **Kristina M. Hill‐Spanik:** resources, supervision, data curation, formal analysis, visualisation, methodology, investigation, writing – review and editing. **Heather Fullerton:** conceptualization, investigation, writing – original draft, funding acquisition, visualisation, methodology, formal analysis, project administration, supervision, resources, writing – review and editing, software.

## Conflicts of Interest

The authors declare no conflicts of interest.

## Supporting information


**Figure S1:** Rarefaction curves of bacterial partial small subunit (SSU) rRNA gene sequences for each macroalgal sample and background water control. Colour represents macroalgal class. Green algae (Ulvophyceae), red algae (Florideophyceae), brown algae (Phaeophyceae), and background water control (blue). Sample with the fewest number of sequences represented by the vertical black line.

**Figure S2:** Venn diagrams of the number of core amplicon sequence variants (ASVs) over the five collection days for each macroalgal species. Core ASVs were identified as those found in all triplicate samples per day.

**Table S1:** Accession information for the macroalgal species used in this study at the Bernice Pauahi Bishop Museum in Honolulu, HI (* indicates voucher specimen from Kuba et al. 2021).

**Table S2:** Summary of temperature and light data collected from HOBO Pendant MX Temperature/Light Loggers placed in the low, mid, and high intertidal bench from 15/5/2023 to 17/5/2023, 2 years after macroalgal samples were collected. Values across tidal zones are reported as ranges, as well as were averaged and their standard deviation was calculated.

**Table S3:** Number of observed amplicon sequence variants (ASVs) of bacterial taxa and Shannon and Simpson's diversity indices for all macroalgal and background water control samples. Shannon and Simpson's diversity indices were chosen to show differences in both richness and evenness, respectively.

**Table S4:** Average of Observed ASVs, Shannon, and Simpson's diversity indicies for all macroalgal classes and seawater controls.

**Table S5:** Summary of all *p*‐values for bacterial diversity indices (observed amplicon sequence variants, Shannon, and Simpson's diversity) amongst macroalgal classes and amongst days for each macroalgal species. Models were performed with a one‐way ANOVA (analysis of variance) followed by a Tukey Honest Significant Differences test. † indicates the use of a Kruskal‐Wallis test followed by a post hoc Dunn's test with Bonferroni‐Holm correction due to violation of ANOVA assumption.

**Table S6:** Summary of PERMDISP (permutation test of multivariate homogeneity of groups dispersions) *p*‐values and PERMANOVA (permutational analysis of variance) results based on Bray‐Curtis dissimilarities of amplicon sequence variants of all factors.

**Table S7:** Taxonomy of the top BLAST search results of the sequences for the top five ASVs identified by SIMPER to be contributing to day‐to‐day dissimilarities for *A. spicifera* , L. dendroidea, and D. sandvicensis shown in Figure 6.

## Data Availability

Sequencing data are available through NCBI Sequence Read Archive (SRA) under study number SUB15260747 (BioProject number PRJNA1259966).
