# Supplementary figures and images for: Examining Short Temporal Changes in Intertidal Macroalgal Microbiomes at 'Ewa Beach, O'Ahu, Hawai'i: Some Hosts Varied While Others Remained Stable
Source: Environ Microbiol Rep. 2026 Apr 6;18(2):e70333. doi: 10.1111/1758-2229.70333 (PMC13052502; doi:10.1111/1758-2229.70333)

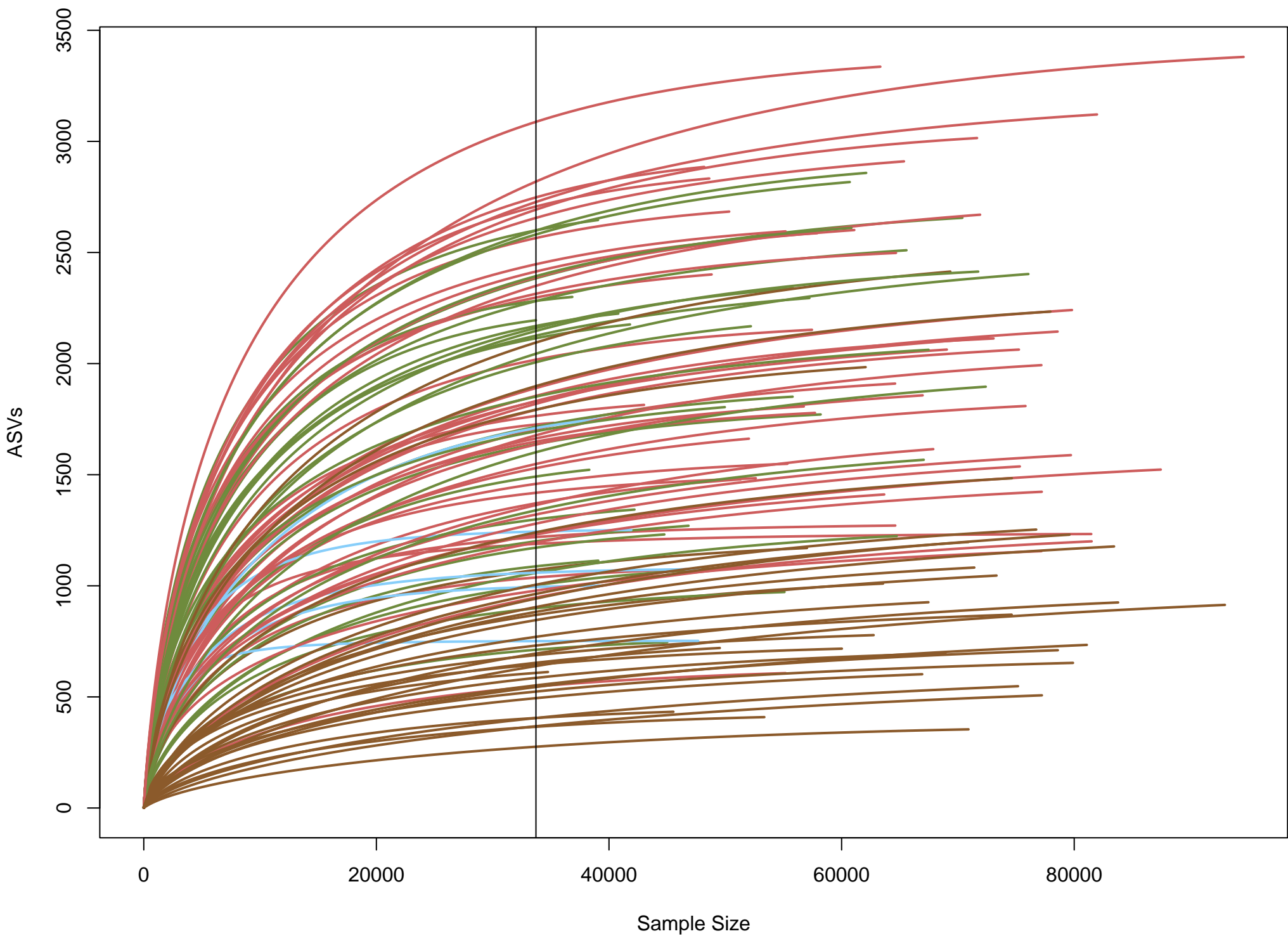

Supplement: Supplementary file 1 — Figure S1: Rarefaction curves of bacterial partial small subunit (SSU) rRNA gene sequences for each macroalgal sample and background water control. Colour represents macroalgal class. Green algae (Ulvophyceae), red algae (Florideophyceae), brown algae (Phaeophyceae), and background water control (blue). Sample with the fewest number of sequences represented by the vertical black line. [file EMI4-18-e70333-s003.pdf]

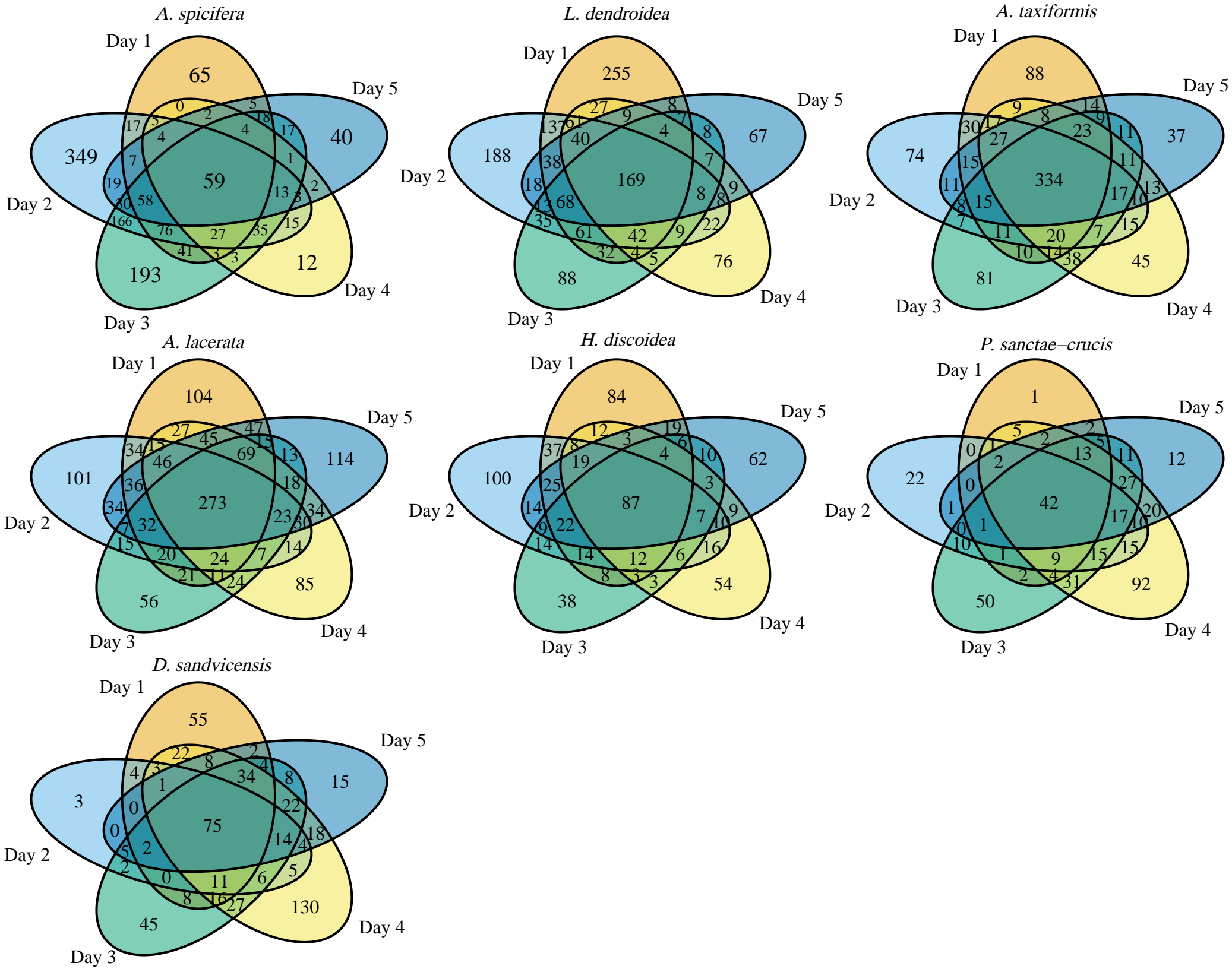

Supplement: Supplementary file 2 — Figure S2: Venn diagrams of the number of core amplicon sequence variants (ASVs) over the five collection days for each macroalgal species. Core ASVs were identified as those found in all triplicate samples per day. [file EMI4-18-e70333-s001.pdf]
